# Integrated bioinformatics analysis for novel miRNAs markers and ceRNA network in diabetic retinopathy

**DOI:** 10.3389/fgene.2022.874885

**Published:** 2022-09-16

**Authors:** Jingru Li, Chaozhong Li, Yulan Zhao, Xinyu Wu, Shuai Yu, Guihu Sun, Peng Ding, Si Lu, Lijiao Zhang, Ping Yang, Yunzhu Peng, Jingyun Fu, Luqiao Wang

**Affiliations:** ^1^ Department of Cardiology, The First Affiliated Hospital of Kunming Medical University, Kunming, China; ^2^ Department of Emergency, The First Affiliated Hospital of Kunming Medical University, Kunming, China; ^3^ Department of Laboratory Animal Science, Kunming Medical University, Kunming, China; ^4^ Department of Endocrinology, The First Affiliated Hospital of Kunming Medical University, Kunming, China

**Keywords:** diabetic retinopathy, biomarker, microarray, bioinformatics analysis, RNA regulatory pathways

## Abstract

In order to seek a more outstanding diagnosis and treatment of diabetic retinopathy (DR), we predicted the miRNA biomarkers of DR and explored the pathological mechanism of DR through bioinformatics analysis.

**Method:** Based on public omics data and databases, we investigated ncRNA (non-coding RNA) functions based on the ceRNA hypothesis.

**Result:** Among differentially expressed miRNAs (DE-miRNAs), hsa-miR-1179, -4797-3p and -665 may be diagnosis biomarkers of DR. Functional enrichment analysis revealed differentially expressed mRNAs (DE-mRNAs) enriched in mitochondrial transport, cellular respiration and energy derivation. 18 tissue/organ-specific expressed genes, 10 hub genes and gene cluster modules were identified. The ceRNA networks lncRNA FBXL19-AS1/miR-378f/MRPL39 and lncRNA UBL7-AS1/miR-378f/MRPL39 might be potential RNA regulatory pathways in DR.

**Conclusion:** Differentially expressed hsa-miR-1179, -4797-3p and -665 can be used as powerful markers for DR diagnosis, and the ceRNA network: lncRNA FBXL19-AS1/UBL7-AS1-miR-378f-MRPL39 may represent an important regulatory role in DR progression.

## Introduction

Diabetic retinopathy (DR) is a complication of diabetes that occurs in specific microvessels. It is also a major cause of preventable blindness among working-age groups, affecting more than 4 million diabetic patients (Simó-Servat et al., 2012; Loukovaara et al., 2015). With the prolongation of the disease time, the lesions of diabetes mellitus (DM) gradually aggravate, so DR patients also have different degrees of retinopathy due to different disease stages. Failure to intervene in DR in a timely manner will result in severe visual impairment in patients (Yin et al., 2020). Therefore, early diagnosis and treatment of DR are important means to prevent retinal deterioration and improve the quality of life of patients (Reichard et al., 1991).

Presently, the most common method of diagnosing DR is to combine a comprehensive ophthalmic examination with the observed clinical characteristics (Solomon et al., 2017). However, the current complex inspection method is not only costly but also has low detection sensitivity, which cannot detect DR at an early stage. Therefore, there is an urgent need for sensitive and rapid biomarkers that can be used for the early diagnosis of DR.

Transcriptomics and microarray analysis are currently commonly used methods for studying the molecular mechanisms of diseases. By detecting gene expression during disease progression, new biomarkers associated with the disease can be identified and potential pathological mechanisms of disease can be explored. Transcriptomics and microarray analysis have been widely used in a variety of diseases, including tumors and DR. (Demircioglu et al., 2019; Youngblood et al., 2019; Carr et al., 2020; Gu et al., 2020; Sun et al., 2020). In addition, competing endogenous RNA (ceRNA) networks can elucidate novel mechanisms of disease development at the level of transcriptional regulation (Salmena et al., 2011). Therefore, combining microarray identification with bioinformatics analysis to find potential novel biomarkers, hub genes and ceRNA regulatory networks in DR will contribute to the in-depth understanding of the pathogenesis of DR, providing new directions and goals for early clinical intervention and treatment of DR.

To explore novel biomarkers with potential DR diagnostic roles and their regulatory mechanisms, this study used the GEO dataset to identify differentially expressed genes (DEGs) in DR. Then, a series of comprehensive bioinformatic analysis methods were employed to screen for DR diagnostic-related miRNA biomarkers and investigate the potential regulatory mechanisms in DR, including functional enrichment analysis, protein-protein interaction (PPI) network construction, tissue/organ-specific expression gene identification, and ncRNA prediction. The combination of mRNA and ncRNA will not only provide a novel diagnostic approach for DR but also deepen the understanding of the pathogenesis of DR. Overall, this work will explore the novel mechanisms of DR progression at the transcriptome level and provide a basis for early clinical intervention and targeted therapy in DR.

## Materials and methods

### Expression profile of miRNA and mRNA in microarray data from patients with diabetic retinopathy

Five microarray datasets containing DR patients were obtained from the National Institutes of Health (NIH)-National Center for Biotechnology Information (NCBI)-Gene Expression Omnibus Database (GEO) (https://www.NCBI.nlm.NIH.gov/gds/). The details of these GEO datasets are shown in Table 1. The same sample—the retina, was used in the original microarray experiments for these datasets, which provided us with comparing the effects of ceRNAs in identical cell types. It needs to be stated that our method is proven and feasible. For example, as an idiomatic rehearsal, we (Wang et al., 2016; Wang et al., 2017; Li et al., 2022) and others (Li et al., 2016) have studied gene expression under pathophysiological conditions.

**TABLE 1:** Information of selected microarray datasets.

GEO accession	Source tissue	Sample		Data	Attribute
		Control	DR		
GSE140959	Human Plasma	4	6	miRNA	Test set
GSE53257	Human Retina	5	6	mRNA	Test set
GSE160308	Human Retina	20	59	miRNA	Validation set
GSE160306	Human Retina	20	59	lncRNA	Validation set
GSE144605	HRMVPC	3	3	mRNA	Validation set

HRMVPC: human retinal microvascular pericytes.

### Functional enrichment analyses

The biological function of the identified target DEGs was assessed using the Database for Annotation, Visualization, and Integrated Discovery version (DAVID, https://david.ncifcrf.gov/) Bioinformatics Resources (v6.8). Briefly, the shared DEGs were imported into DAVID, followed by Gene Ontology (GO) and Kyoto Encyclopedia of Genes and Genomes (KEGG) functional enrichment analysis. GO analysis assessed the biological processes (BPs), molecular functions (MFs) and cellular components (CCs) involved in genes, and KEGG assessed the regulatory pathways involved in genes.

### Protein-protein interaction analysis

Protein-protein interaction (PPI) networks were conducted by the STRING database (https://string-db.org/). A moderate confidence value of 0.4 was used as the screening threshold. Next, in order to obtain the hub genes, a visualized PPI regulatory network was constructed using Cytoscape software (version 3.4.0), (Shannon et al., 2003). The Minimal Common Oncology Data Elements (MCODE) plugin was employed to identify highly correlated gene clusters and obtain cluster scores (filtering criteria: degree cutoff = 2; node score cutoff = 0.2; k-core = 2; maximum depth = 100), and the CytoHubba plugin was used to extract hub genes from the PPI network (Chin et al., 2014). In the process of identifying hub genes, five algorithms of degree, maximum cluster centrality (MCC), maximum neighborhood component (MNC), maximum neighborhood component density (DMNC) and clustering coefficient were used and finally obtained the top 10 hub genes (Yang et al., 2019; Luan et al., 2020).

### Identification of tissue/organ-specific expressed genes

To understand whether these DEGs are distributed in specific tissues/organs, the online tool BioGPS (BioGPS, http://biogps.org/) was used to analyze the tissue/organ-specific localization of DEGs (Wu et al., 2009). The specific screening criteria are as follows: (Simó-Servat et al., 2012) the projected amount of transcripts in a single organ system, the expression value of which is greater than 10 times the median value of all organ systems, (Loukovaara et al., 2015) the expression level of transcripts in the second most abundant tissue is not equal more than one-third of the expression in the highest expressing tissue (Wu et al., 2009). After two screenings, the eligible genes were considered as tissue-specific expressed genes.

### Prediction of target miRNAs

RNA22 (https://cm.jefferson.edu/rna22/) (Loher and Rigoutsos, 2012), miRWalk (http://mirwalk.umm.uni-heidelberg.de/) (Dweep et al., 2014) and mirDIP (http://ophid.utoronto.ca/mirDIP/) (Tokar et al., 2018) are three online miRNA-mRNA interaction prediction databases. We used these three databases for upstream miRNA prediction of hub genes and selected miRNAs that co-existed in at least three databases as target miRNAs. In view of the interaction between messenger RNA and miRNA, we used Cytoscape to establish an mRNA-miRNA co-expression network.

### Construction of ceRNA networks

StarBase (Yang et al., 2011) (version 3.0) (http://starbase.sysu.edu.cn/index.php), DIANA-LncBase (Karagkouni et al., 2020) (version 3.0, https://diana.e-ce.uth.gr/lncbasev3), RNA22 (Loher and Rigoutsos, 2012) (https://cm.jefferson.edu/rna22/Interactive/) and lncACTdb (Wang et al., 2021) (version 3.0, http://bio-bigdata.hrbmu.edu.cn/LncACTdb/index.htm) were used to predict lncRNAs that interacted with the selected miRNAs (Li et al., 2014). The shared lncRNAs in the prediction results of the four databases were regarded as candidate target lncRNAs, and the candidate target lncRNAs were further validated using the GEO dataset. The ceRNA network was visualized using Cytoscape to describe the interactions among mRNAs, miRNAs, and lncRNAs.

### Statistical analysis of microarray data

Two-tailed Student’s t-test was used to analyze differences between groups. The R statistical analysis package (version 3.5.3) performed statistical analysis of the data. An alpha value of *p* < 0.05 was measured in this study and *p* < 0.05 was considered statistically significant.

## Result

### Screening of diagnostic markers in human plasma of diabetic retinopathy

**FIGURE 1:**
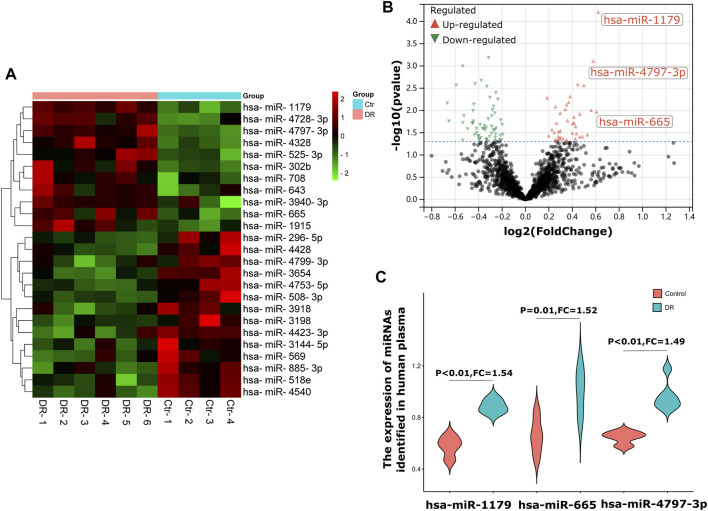
Screening of diagnostic markers of human plasma in DR. **(A)**. A heatmap corresponding to the expression profile of the top 25 DE-miRNAs in human plasma in the GSE140959 dataset as determined based on *p* values. Red rectangles represent high expression, and green rectangles represent a low expression. **(B)**. A volcano plot corresponding to the expression profile of miRNAs in plasma in the GSE140959 dataset. The red plots represent upregulated miRNAs, the black plots represent nonsignificant miRNAs, and the green plots represent downregulated miRNAs. **(C)**. The Violin charts show the fold change of the top three upregulated miRNAs identified in human plasma. DE-miRNAs: differentially expressed miRNAs; DR: Diabetic Retinopathy; FC: Fold Change.

In this study, the human plasma GEO dataset GSE140959 was selected as the test dataset for screening DR biomarkers. A total of 10 samples was collected in the GSE140959 dataset: 6 of them were plasma samples from diabetic (DM) type II DR (DMII-DR) patients and 4 of them were plasma samples from DM II non-DR (DMII-NDR) patients. As shown in the heatmaps and volcano plots in Figures 1A,B total of 98 differentially expressed miRNAs (DE-miRNAs) (40 upregulated and 58 downregulated) were identified in the GSE140959 dataset. Given the evidence that miRNAs are readily available and have good diagnostic properties, we propose to use the top three miRNAs with the highest increase in expression as biomarkers. Among the miRNAs upregulated in DR plasma, hsa-miR-1179 (FC = 1.54, *p* < 0.01), hsa-miR-4797-3p (FC = 1.52, *p* = 0.01) and hsa-miR- 665 (FC = 1.49, *p* < 0.01) were the top 3 with the largest changes in expression and were statistically significant (Figure 1C). Therefore, based on the GSE140959 database samples, we hypothesized that hsa-miR-1179, -4797-3p and -665 may be potential biomarkers for early diagnosis of DR. Similar to this study, it has been previously demonstrated that miRNAs with the greatest differential expression in patient plasma compared to normal individuals are efficient biomarkers for disease diagnosis (Tian et al., 2016).

### Identification of DE-mRNAs in the human retina

**FIGURE 2:**
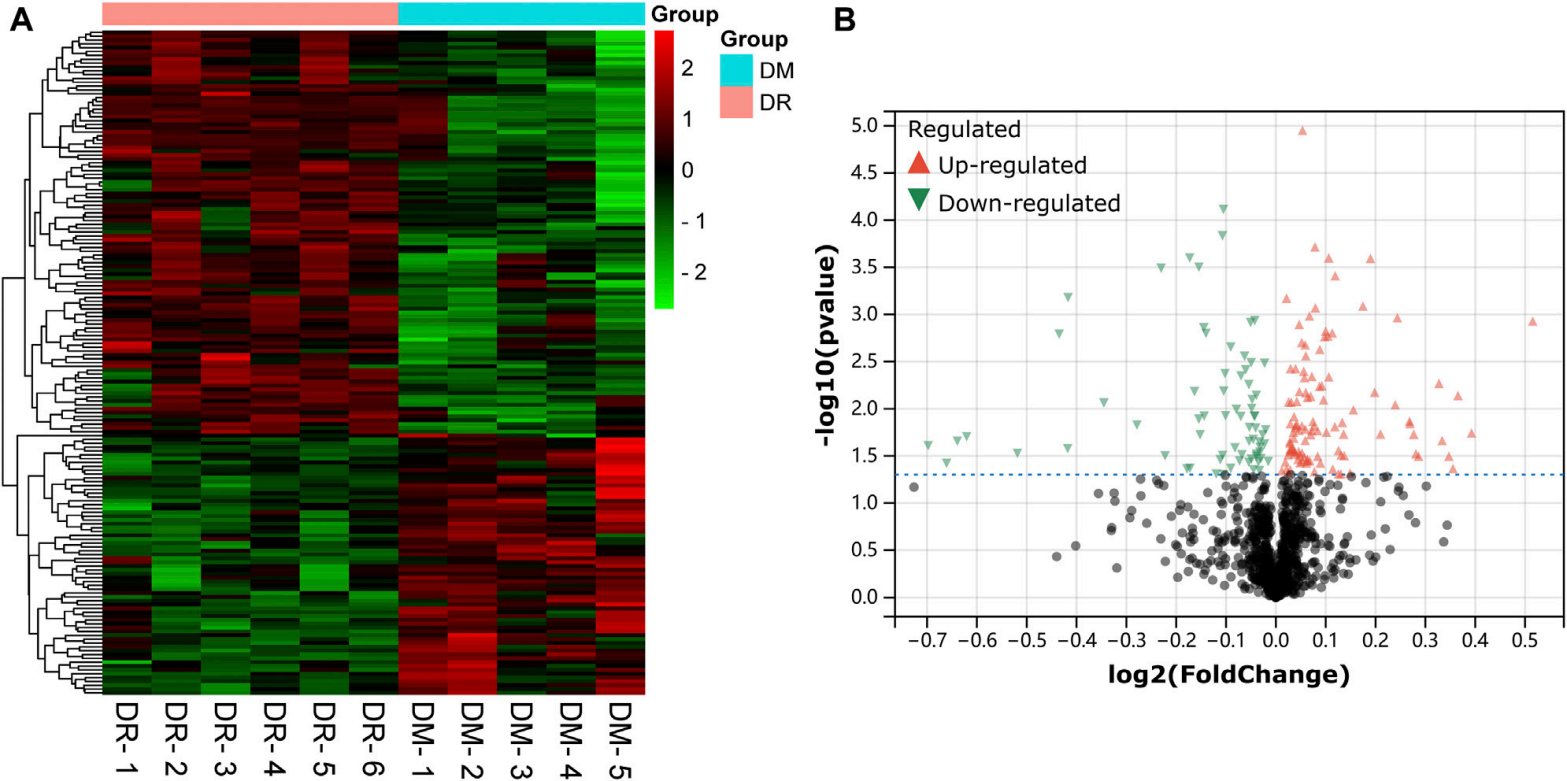
Detection of differentially expressed mRNAs (DE-mRNAs) of human retina in the GSE53257 dataset. **(A)**. An expression heatmap corresponding to the expression profile of mRNAs in the human retina. **(B)**. A volcano plot corresponding to the expression profile of mRNAs in the human retina. The red plots represent upregulated genes, the black plots represent nonsignificant genes, and the blue plots represent downregulated genes. DE-mRNAs: differentially expressed mRNAs.

A dataset of retinal samples from diabetic patients—GSE53257 was used to extract differentially expressed mRNAs. GSE53257 has a total of 11 samples: 6 samples were from the retina of diabetic patients with DR, and five samples were from the retina of non-DR diabetics. As shown in the cluster heat map and volcano plots results in Figures 2A,B total of 178 differentially expressed mRNAs (DE-mRNAs) (108 upregulated and 70 downregulated) were identified in the GSE53257 dataset.

### Pathway enrichment analyses

**FIGURE 3:**
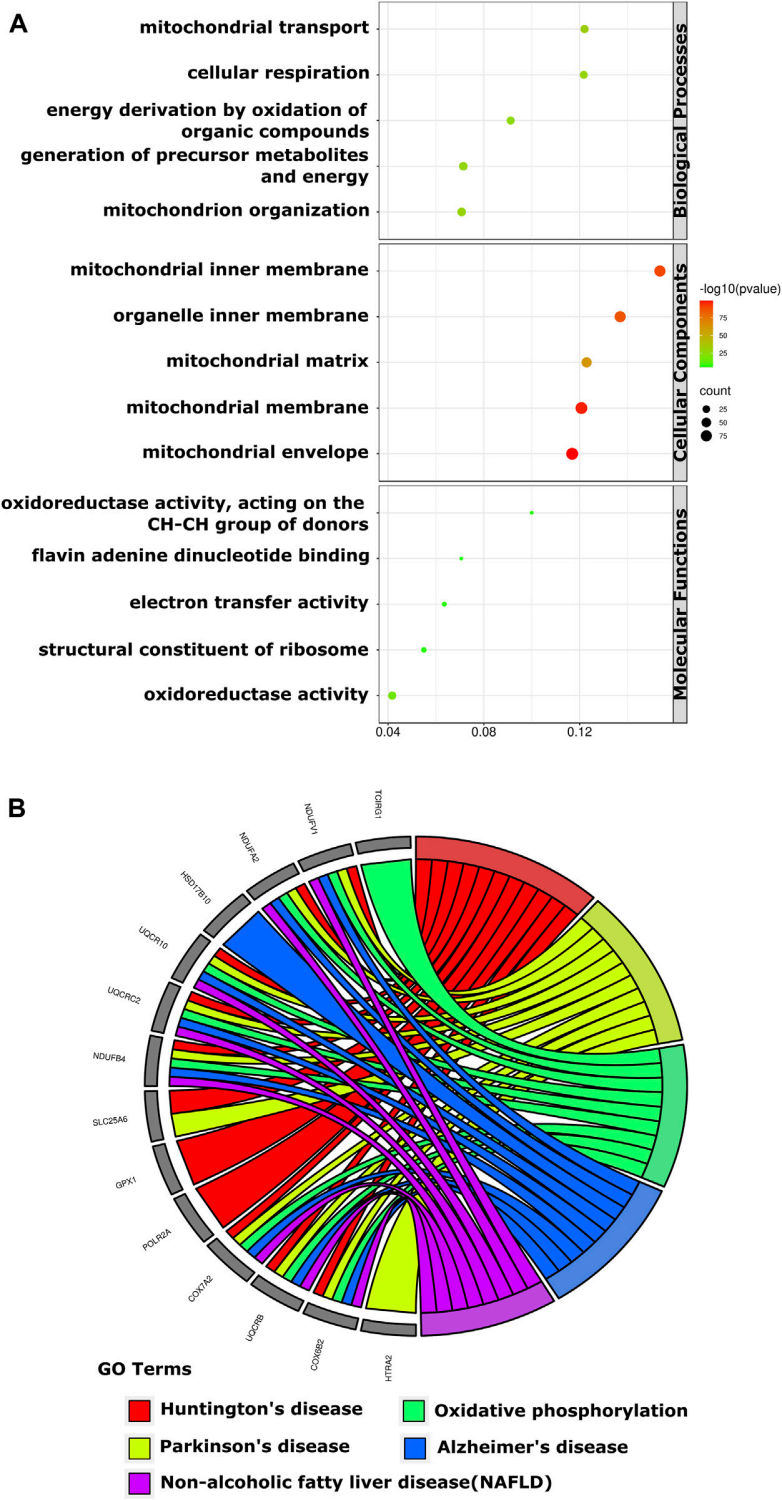
The GO enrichment and KEGG pathways of DE-mRNAs of the human retina in the GSE53257 dataset. **(A)**. The bubble plot shows the top five GO enriched terms in BP, CC, and MF. The most significant BP involved in mitochondrial transport, cellular respiration and energy derivation by oxidation of organic compounds, CC involved in mitochondrial inner membrane, organelle inner membrane and mitochondrial matrix, and MF involved in oxidoreductase activity, Flavin adenine dinucleotide binding and electron transfer activity. **(B)**. The chord plot shows the most enriched KEGG pathways of DE-mRNAs. The most significant KEGG pathways are involved in Huntington’s, Parkinson’s and Alzheimer’s disease. DE-mRNAs: differentially expressed mRNAs; GO: Gene Ontology; BP: biological process; CC: cellular components; MF: molecular function; KEGG: Kyoto Encyclopedia Genes and Genomes; The screening criteria for significant enriched biological processes and pathways were Q < 0.05. The Q value is the adjusted *p*-value.

The result of GO analysis disclosed that these DE-mRNAs were interrelated with diverse biological processes, molecular functions, and cellular components. The top three biological processes those DE-mRNAs were comprised of: mitochondrial transport, cellular respiration, and energy derivation by oxidation of organic compounds; The top three significant cellular components related to those mRNAs were: mitochondrial inner membrane, organelle inner membrane, and mitochondrial matrix; The three most significant molecular functions in which the DE-mRNAs participated were: oxidoreductase activity, flavin adenine dinucleotide binding and electron transfer activity (Figure 3A). KEGG pathway analysis showed those DE-mRNAs to be significantly enriched in huntington’s, parkinson’s, and alzheimer’s disease (Figure 3B).

### The immune/haematologic system is the primary tissue in which DE-mRNAs are enriched

A total of 18 tissue/organ-specific expressed genes were identified by BioGPS (Table 2). After analysis, we found that the great majority of these DE-mRNAs were specifically expressed in the immune/haematologic system, with a total of 11 genes (11/18, 61.11%). The second organ-specific expressed system was the digestive system, which included 4 genes (4/18, 22.22%). This was followed by adipose tissue (2/18, 11.11%), and circulatory system (1/18, 5.55%).

**TABLE 2:** Distribution of tissue/organ-specific expressed genes identified by BioGPS.

System/Organ	Genes	Counts
Immune/Haematologic	OXSM, WARS2, ABCB6, ALAS2, ABCB10, PPOX, ACSM3, TOMM22, MRPL32, FECH, HTRA2	11
Digestive system	GLS2, SUOX, APOA1, ECHS1	4
Adipose tissue	GPD1, DLAT	2
Circulatory system	PLA2G15	1

### MRPL39 is a hub gene in diabetic retinopathy and is specifically expressed in the immune system

**FIGURE 4:**
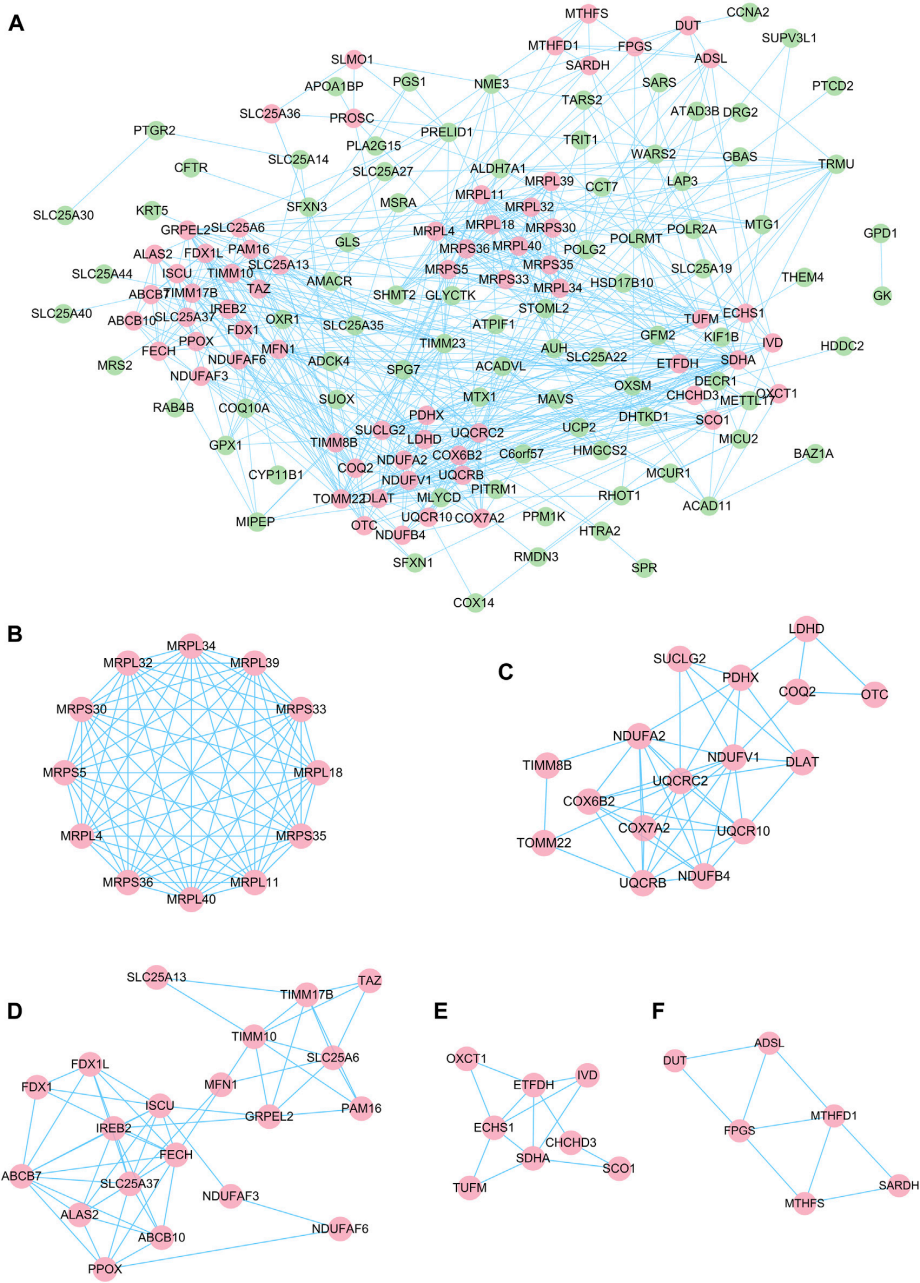
PPI network of DE-mRNAs and six cluster modules extracted by MCODE. **(A)**. The interaction network between proteins coded by DE-mRNAs was comprised of 170 nodes and 626 edges. Each node represents a protein, while each edge represents one protein-protein association. The smaller the value of Q is, the larger the shape size. Six cluster modules were extracted by MCODE. **(B)**. Cluster 1 had the highest cluster score (score: 12, 12 nodes and 66 edges), followed by cluster 2 **(C)** (score: 6.4, 16 nodes and 48 edges), cluster 3 **(D)** (score: 6, 20 nodes and 57 edges), cluster 4 **(E)** (score: 3.714, 8 nodes and 13 edges) and cluster 5 **(F)** (score: 3.6, 6 nodes and 9 edges). The color of the node represents the expression level of the gene in the dataset GSE53257: pink represents upregulation, green represents downregulation.

The STRING online tool was used in this study to analyze the interactions between proteins encoded by DEGs. The results of the PPI network showed that the interactions between DEGs form a complex network containing 170 nodes and 626 edges (Figure 4A). The MCODE plug-in was used to identify the gene clusters in the PPI network and finally five modules were obtained, as shown in Figures 4B–F. Cluster 1 has the highest cluster score (score: 12, 12 nodes, and 66 edges), followed by cluster 2 (score: 6.4, 16 nodes, and 48 edges), and cluster 3 (score: 6, 20 nodes, and 57 edges), cluster 4 (score: 3.714, 8 nodes and 13 edges) and cluster 5 (score: 3.6, 6 nodes and 9 edges). The results of the MCODE analysis reveal that the genes in these five clusters are key genes in the progression of DR with potential regulatory roles. Then to understand the main biological processes involved in the clusters, we performed GO/KEGG analysis on 12 genes in the highest scoring cluster 1. The analysis showed that the main biological process involved in cluster 1 is mitochondrial translation, and the main pathway involved in cluster 1 is the ribosomal pathway. The results of GO/KEGG analysis of cluster 1 are shown in Supplementary Table S1.

**FIGURE 5:**
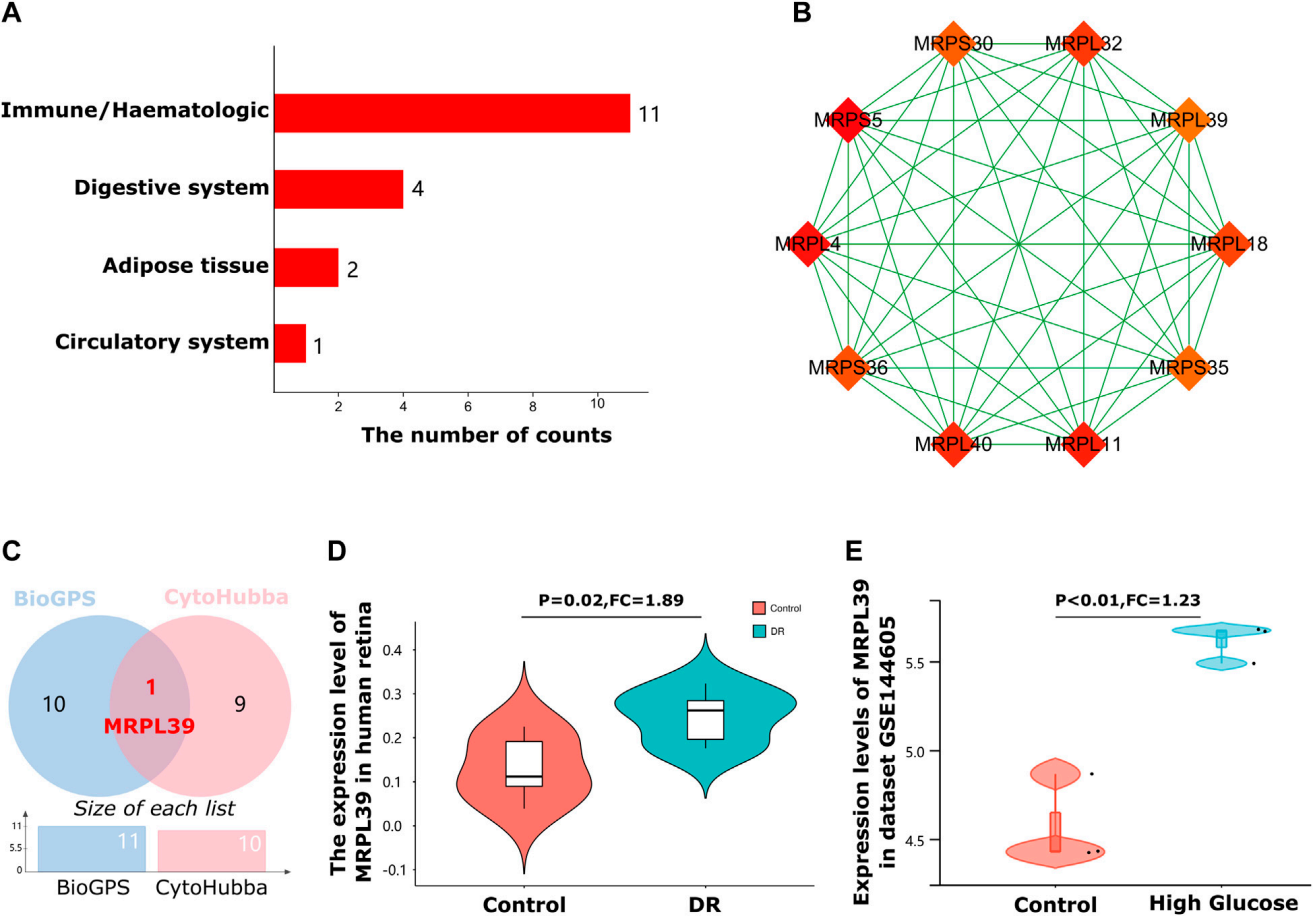
Immune system-specific expressed hub genes identification. **(A)**. The bar graph shows that most of these DE-mRNAs were specifically expressed in the haematologic/immune system (11/18, 61.11%). **(B)**. Cluster plots represent the top 10 hub genes identified by Cytohubba. **(C)**. A Venn diagram of immune system-specific expressed hub genes of the 2 databases. **(D)**. The Violin charts represent the expression of MRPL39 in the human retina. **(E)**. The Violin charts represent the expression of MRPL39 in dataset GSE144605.

Next, we further identified tissue-specific and hub genes. BioGPS analysis showed that these DE-mRNAs were mainly specifically expressed in the haematologic/immune system (Figure 5A). In addition, the results of the five algorithms of CytoHubba were intersected, and the top ten hub genes were finally identified (Figure 5B). Details of these hub genes are shown in Table 3. These genes take over large scores in the PPI network, which indirectly suggests that they may play a significant role in the pathogenesis of DR.

**TABLE 3:** 10 hub genes identified by five algorithms of CytoHubba.

Gene symbol	Description	Log2FC	*p*-value	Regulation
MRPS5	mitochondrial ribosomal protein S5	−0.22655	0.030776	Down
MRPL4	mitochondrial ribosomal protein L4	−0.16126	0.009908	Down
MRPL11	mitochondrial ribosomal protein L11	−0.16848	0.001218	Down
MRPL40	mitochondrial ribosomal protein L40	0.247277	0.013626	Up
MRPL32	mitochondrial ribosomal protein L32	−0.14053	0.011982	Down
MRPL18	mitochondrial ribosomal protein L18	0.416695	0.048889	Up
MRPS36	mitochondrial ribosomal protein S36	1.1534	0.031844	Up
MRPS30	mitochondrial ribosomal protein S30	0.093226	0.028237	Up
MRPS35	mitochondrial ribosomal protein S35	−2.12334	0.022005	Down
MRPL39	mitochondrial ribosomal protein L39	0.919154	0.018584	Up

As a common diabetes-related autoimmune disease, an in-depth understanding of the immune-related mechanisms of DR is crucial for the treatment of DR. Therefore, to explore genes with potential immune regulatory roles in diabetic retinopathy, we intersected 10 hub genes of DR with 11 genes that are specifically expressed in the immune system (Figure 5C). The results of the intersection showed that MRPL39 was a gene with both a pivotal position in DR and immune system-specific expression properties. Meanwhile, MRPL39 was upregulated in DR retinal tissue (FC = 1.89, *p* = 0.02) (Figure 5D). To further confirm the reliability of MRPL39 expression level changes in diabetic retinopathy, dataset GSE144605 was used to validate MRPL39 expression. Dataset GSE144605 is an array dataset containing sequencing data from high glucose-treated human retinal microvascular pericytes (HRMVPC). data from three high glucose cultured HRMVPC samples and data from three normal glucose cultured HRMVPC samples were used in this study. The validation results showed that MRPL39 also showed high expression in dataset GSE144605 which was statistically significant. The expression levels of MRPL39 in the data set GSE144605 are shown in Figure 5E. All these suggest that MRPL39 may be a key target for early intervention and treatment of DR.

### hsa-miR-378f and -6849-5p are the upstream regulatory miRNAs of MRPL39

**FIGURE 6:**
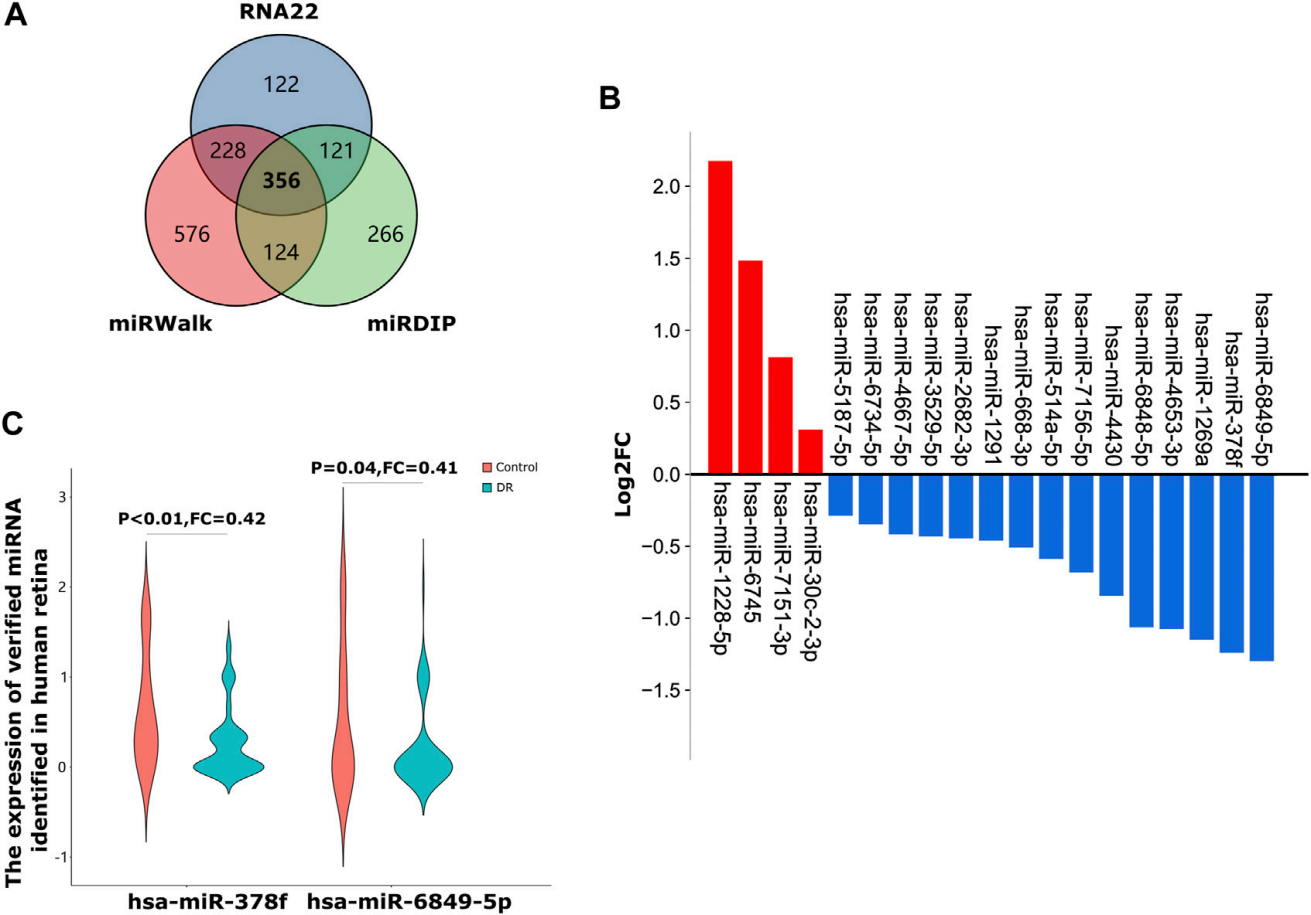
A co-expressed network of MRLP39 and its target miRNAs. **(A)**. A Venn diagram of shared target miRNAs of MRPL39 of the three databases. **(B)**. The bar graph shows that 19 out of 356 shared miRNAs verified by the GSE160308 dataset, including 4 up-regulated and 15 down-regulated. The red bar graph means upregulation and the blue means downregulation. **(C)**. The Violin charts represent the expression of hsa-miR-378f and hsa-miR-6849-5p identified in biofluids-plasma.

We used three online miRNA databases, RNA22, miRWalk and miRDIP, to predict target miRNAs of MRPL39 (Supplementary Table S2). Finally, we obtained 356 shared target miRNAs of MRPL39 (Figure 6A). We then performed GSE160308: retinal samples from 59 DR patients and 20 non-DR DM patients to verify 356 shared target miRNAs. Finally, we obtained 19 shared miRNAs through validation on the GSE160308 dataset, of which 4 were up-regulated and 15 were down-regulated in DR retinal tissues (Figure 6B). According to the interaction relationship between miRNA-mRNA (the levels of miRNA and mRNA tend to be negatively correlated), we selected the top 2 down-regulated miRNAs in the retina of DR patients for further analysis. Among the down-regulated target miRNAs, the 2 miRNAs with the greatest changes were hsa-miR-378f (FC = 0.42, *p* < 0.01) and hsa-miR-6849-5p (FC = 0.41, *p* = 0.04) (Figure 6C). Therefore, we initially constructed a co-expression network of MRPL39 and miRNAs: hsa-miR-378f/MRPL39 and hsa-miR-6849-5p/MRPL39.

### lncRNA FBXL19-AS1/UBL7-AS1/miR-378f/MRPL39 is a potential regulatory ceRNA network in diabetic retinopathy

**FIGURE 7:**
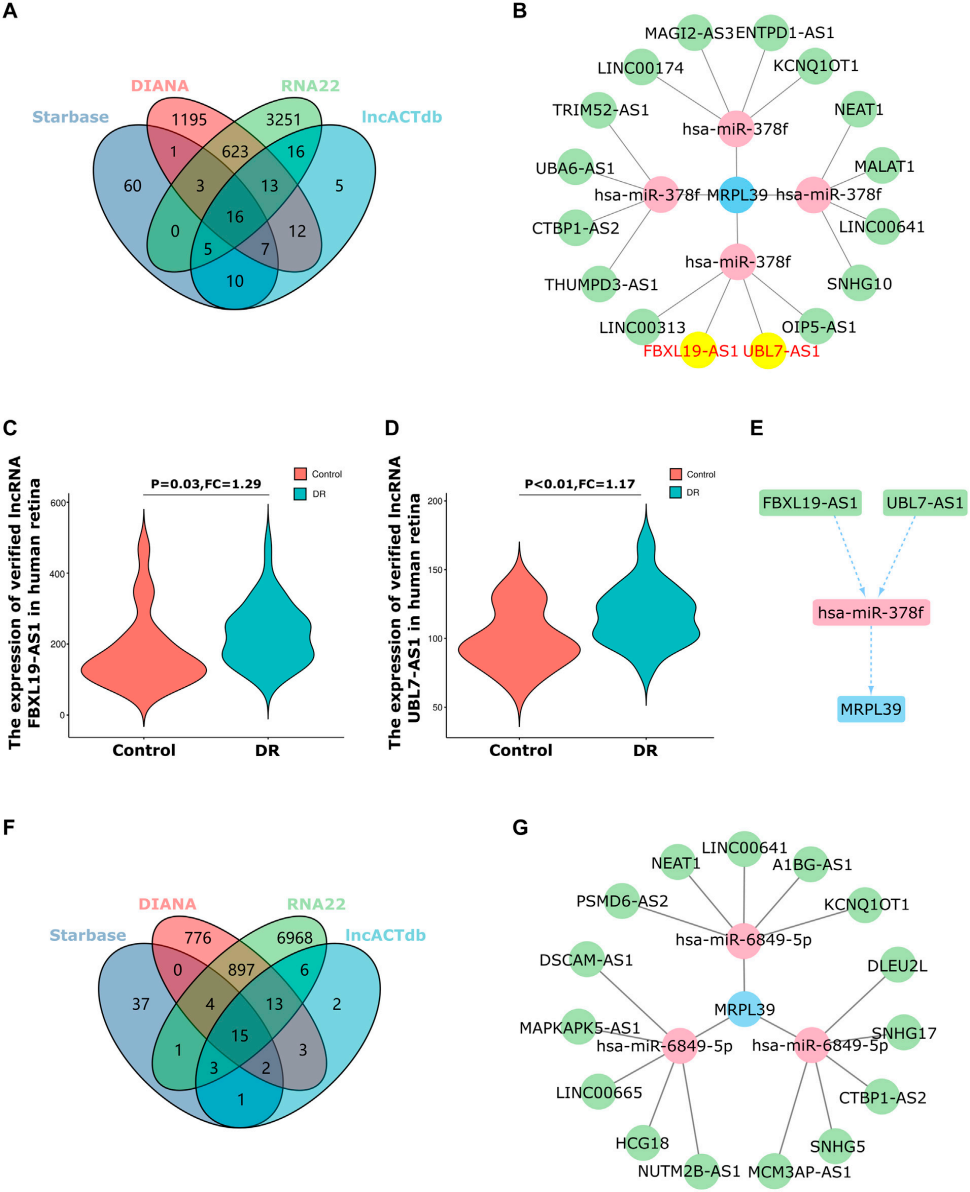
The ceRNA networks construction. **(A–E)**. ceRNA network of hsa-miR-378f-MRPL39: **(A)**The Venn diagram of 16 shared target lncRNAs of hsa-miR-378f of the 4 datasets; **(B)**. Then five out 16 shared lncRNA were verified by GSE160306 dataset, including 2 upregulation (FBXL19-AS1 and UBL7-AS1) and three downregulation (KCNQ1OT1, LINC00641 and MALAT1); **(C–D)**. The Violin charts represent the expression of FBXL19-AS1 and UBL7-AS1; **(E)**. Hance, the ceRNA network was constructed: lncRNA-FBXL19-AS1/miR-378f/MRPL39 and lncRNA-UBL7-AS1/miR-378f/MRPL39. **(F)**. The Venn diagram of 15 shared target lncRNAs of hsa-miR-6849-5p of the 4 datasets; **(G)**.15 shared lncRNA could not be verified by the GSE160306 dataset.

Be universally known that miRNAs can induce the silencing of target genes and inhibit gene expression by pairing with mRNAs. However, upstream lncRNAs can also inhibit miRNA function by binding to miRNA response elements, thereby exerting target gene regulation. The network of non-coding RNAs interacting with miRNAs to exert regulatory effects on target genes is termed the competitive endogenous RNA (ceRNA) network (Salmena et al., 2011). Next, we used the online database, Starbase 3.0, DIANA, RNA22 and lncACTdb, to predict the lncRNAs that interact with hsa-miR-378f and hsa-miR-6849-5p (Supplementary Table S3–S4). We then validated 16 shared target lncRNAs in the GSE160306 dataset, which included 59 retinal tissues from DR patients and 20 retinal tissues from DM patients without DR. Ultimately, we obtained that five out of 16 shared lncRNAs were verified by the GSE160306 dataset, including 2 up-regulation and three downregulation in DR retina. Based on the lncRNA-miRNA-mRNA pathway hypothesis, we selected up-regulated lncRNAs in the retinas of DR patients for further analysis. Among the upregulated target lncRNAs, the top 2 changed lncRNAs were lncRNA FBXL19-AS1 (FC = 1.29, *p* = 0.03) and lncRNA UBL7-AS1 (FC = 1.17, *p* < 0.01). Thus, the ceRNA network was constructed: lncRNA FBXL19-AS1/miR-378f/MRPL39 and lncRNA UBL7-AS1/miR-378f/MRPL39 (Figures 7A–E). For the hsa-miR-6849-5p/MRPL39 pair, there is no lncRNA in common with the 15 shared lncRNAs in the GSE160306 dataset, and a complete ceRNA network cannot be constructed (Figures 7F,G). Hence, we propose the following lncRNA-miRNA-mRNA pathway: lncRNA FBXL19-AS1/miR-378f/MRPL39 and lncRNA UBL7-AS1/miR-378f/MRPL39; they may be involved in important pathways in DR regulatory mechanisms and occupy a remarkable position.

## Discussion

As a high incidence of microvascular complications of diabetes, the hazard of blindness in DR patients is 25 times higher than that in non-diabetic patients (Wykoff et al., 2021). Due to the insufficiency of clear early diagnostic indicators and insidious symptoms, DR patients often lose the best time for treatment, leading to an inferior prognosis. Therefore, finding more sensitive and concrete diagnostic markers has far-reaching significance for improving the prognosis of DR patients. With the advancement of modern bioassay and sequencing technologies, the genetic information enriched in atrial fluid, tear fluid, plasma and other body fluids can also be detected and is increasingly crucial for the diagnosis and treatment of diseases.

In the first part of the study, we aimed to obtain miRNA biomarkers with diagnostic potential from the plasma of DR patients. In the GSE140959 dataset of human plasma samples, we identified 40 up-regulated miRNAs. Due to the good diagnostic properties of plasma miRNAs (non-invasive collection and faster changes than protein-level DR markers), we defined hsa-miR-1179, -4797-3p and -665, the top three miRNAs with the largest upregulated changes in plasma of DR patients, as potential biomarkers for DR diagnosis. Liu et al.'s study showed that PDR patients had higher levels of hsa-miR-1179 than NPDR patients. The areas under the receiver operating characteristic (ROC) curve of hsa-miR-1179 signature was 0.873. Their data suggested that serum hsa-miR-1179 has the potential to be sensitive, cost-effective biomarkers for the early detection of DR. Identical to this research, our study found that hsa-miR-1179 was upregulated in the DR plasma (Qing et al., 2014). miR-665-3p affect oxygen-glucose deprivation-induced inflammation in microglial cells (Xiang et al., 2021). hsa-miR-665 plays an important role in the development of critical limb ischemia in type 2 diabetic patients (Cheng et al., 2018). However, there has been no relative study on hsa-miR-4797-3p in DR. Therefore, based on the results of our analysis, we concluded that hsa-miR-1179, -4797-3p and -665 could be used as novel biomarkers for DR diagnosis.

Furthermore, aberrant gene expression is commonly associated with disease progression, including DR. Therefore, in an attempt to elucidate the regulatory mechanisms in DR progression, we further used the GEO dataset to explore the pathogenesis of DR. After the identification of hub genes and immune tissue-specific expression genes for 178 DEGs in the dataset GSE53257, we obtained MRPL39, which has both immune system-specific expression properties and is a hub gene for DR.

MRPL39 is a member of the mitochondrial ribosomal proteins (MRPs) family and is mainly involved in the translation of the oxidative phosphorylation complex subunit encoded by mitochondrial DNA (Gopisetty and Thangarajan, 2016; Graack and Wittmann-Liebold, 1998; O'Brien, 2003). Studies have shown that abnormal expression of MRPs will lead to mitochondrial translation dysfunction and damage to the oxidative respiratory chain, thereby causing metabolic disorders in host cells (O'Brien et al., 2005; Sylvester et al., 2004). In addition, some MRPs also play the role of apoptosis-inducing factors. In the basal metabolism of cells, MRPs regulate cell growth and apoptosis through the intrinsic pathway of apoptosis (Huang et al., 2020). MRPL39 also has a role in later stages of mitochondrial ribosome assembly (De Silva et al., 2013), where it may interfere with RNA binding, acquire transmembrane domains and function in the tissue specificity of mitochondrial DNA disease mutations (Spirina et al., 2000). Currently, many studies have demonstrated that MRPL39 plays a part in a variety of cancers (Kim et al., 2017; Xu et al., 2021), but the investigation of MRPL39 in diabetic retinopathy remains insufficient. However, our findings suggest that MRPL39 is likely to be a key gene in DR progression and is associated with the immune mechanism of DR.

ceRNAs are the general term for transcripts that can bind to miRNAs and exert regulatory effects on target genes at the post-transcriptional level. The interactions between ceRNAs and miRNAs form a ceRNA network with regulatory functions (Qi et al., 2015). Therefore, in order to elucidate the upstream drivers of the immune-related hub gene MRPL39 and to elucidate the pathogenesis of DR at the transcriptome level, we proposed to construct miRNA-MRPL39 co-expression pairs and ceRNA networks of MRPL39. The target miRNAs of MRPL39 were obtained by prediction. Then, the predicted miRNAs were validated using the dataset GSE160308, and based on the negative regulatory relationship of miRNA-mRNA pairs, two miRNAs were finally obtained that were down-regulated in the retinal tissue of DR patients: hsa-miR-378f and hsa-miR-6849-5p. Several scholars have identified that miR-378f plays a role in colorectal cancer (Sahu et al., 2019) and ischemic stroke thrombosis (Kim et al., 2021). For example, Kota et al. showed that miR-378f was significantly downregulated in primary colorectal cancer and colorectal cancer (CRC) liver metastasis and that miR-378f inhibited proliferation and metastasis of CRC cells and promoted apoptosis of CRC cells. This suggests that miR-378f could be employed as a biomarker for tumor diagnosis and provide a novel strategy for CRC treatment (Sahu et al., 2019). However, studies on miR-378f and miR-6849-5p in DR are still lacking. Although the current studies on miR-378f and miR-6849-5p in DR are still lacking, the above studies also hint us a potential role of miR-378f and miR-6849-5p in the regulation of DR.

**FIGURE 8:**
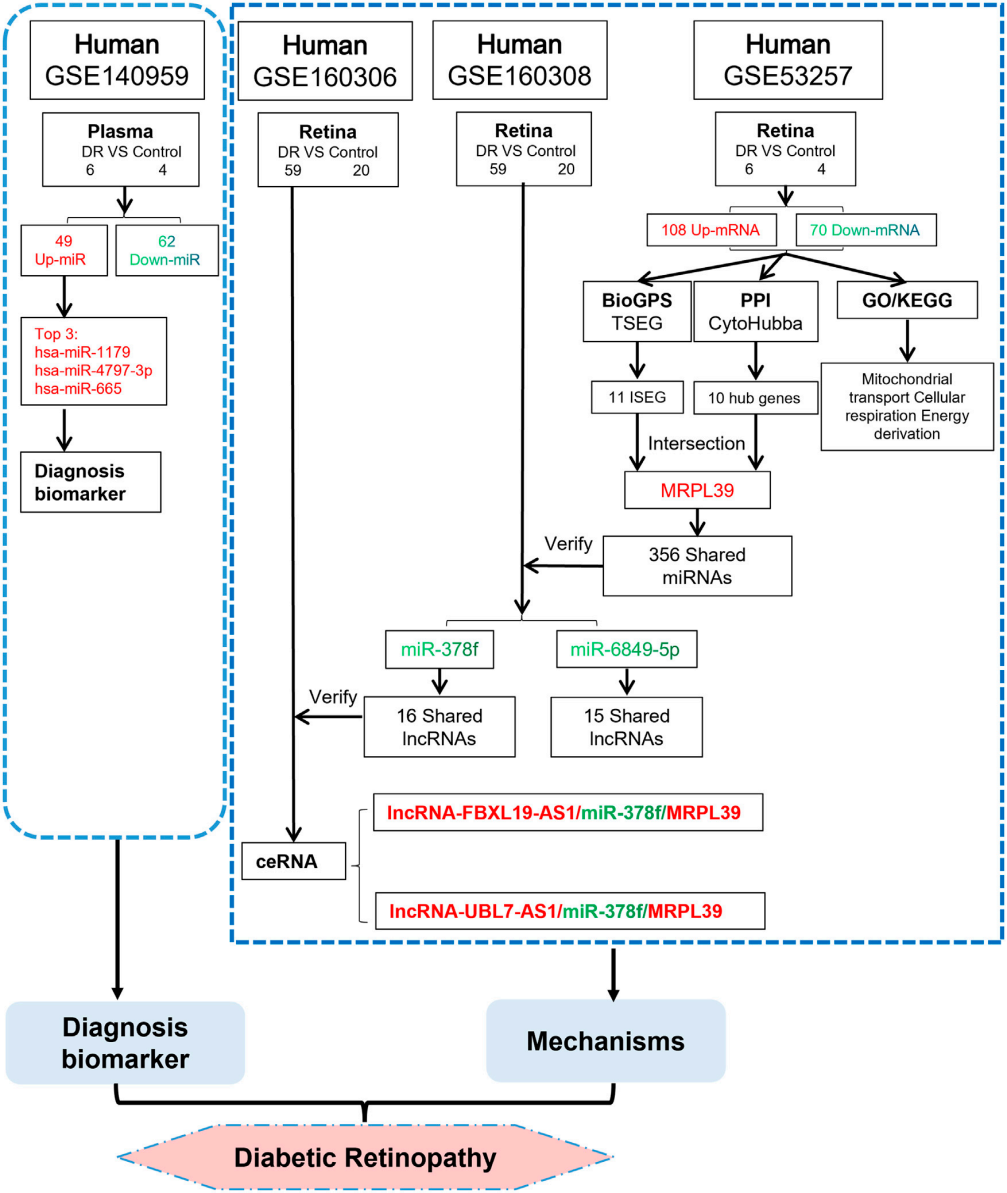
We propose a novel ceRNA network. TSEG: Tissue/organ-Specific Expressed Gene; ISEG: Immune Specific Expressed Gene. The red word means upregulation and the green word means downregulation.

After obtaining the upstream regulatory miRNAs of MRPL39, we further predicted the lncRNAs that interact with miRNAs based on the ceRNA hypothesis and validated the lncRNAs using the GSE160306 dataset. The lncRNAs with increased expression in the dataset GSE160306 were used for validation of the prediction results. Of the lncRNAs obtained for miR-378f, two upregulated lncRNAs were validated in the dataset GSE160306: lncRNA FBXL19-AS1 and lncRNA UBL7-AS1. Numerous studies have examined the role of FBXL19-AS1 and UBL7-AS1 in cancer, but analysis of them in DR remains insufficient. For example, the lncRNA FBXL19-AS1 can play a regulatory role in the proliferation, migration and invasion of osteosarcoma cells through sponge binding to miR-346 (Pan et al., 2018) and promotes breast cancer cells proliferation and invasion *via* acting as a molecular sponge to miR-718 (Ding et al., 2019). lncRNA UBL7-AS1, an immune-related long non-coding RNA, was a prognostic subtype and provided new insights for cervical cancer immunotherapy (Liu et al., 2021). Ultimately, we constructed a new working model (Figure 8) and proposed that lncRNA FBXL19-AS1/miR-378f/MRPL39 and lncRNA UBL7-AS1/miR-378f/MRPL39 may be RNA regulatory pathways with potential regulatory roles in DR progression.

Non-coding RNAs have attracted attention for their diversity of species and rich regulatory functions. And with the advancement of genetic testing technology, the approach of transcript sequencing to discover variations in gene expression in disease states and explore biomarkers with potential disease diagnostic roles has been a current trend in medical treatment (Meder et al., 2011). The abnormally high expression of hsa-miR-1179, -4797-3p and -665 in transcript sequencing data from DR patients suggests that they are likely to be effector molecules in DR progression; and the human plasma source of these sequencing data further enhances the potential of hsa-miR-1179, -4797-3p and -665 as biomarkers for early clinical diagnosis of DR. Not only that, ncRNA as a therapeutic target for diseases has gradually begun to be experimentally confirmed, targeting to enhance or inhibit ncRNA can alleviate pathological damage (Han et al., 2014; Liu et al., 2014). lncRNA FBXL19-AS1/miR-378f/MRPL39 and lncRNA UBL7-AS1/miR-378f/MRPL39 serve as potential regulatory networks for DR. By overexpressing or knocking out lncRNA or target genes, the biological regulatory effects of ceRNA networks in DR can be demonstrated. Therefore, the reproducible validation of the findings of this study by using standardized ncRNA research workflows and accurate patient cohort clinical trials will further advance the realization of hsa-miR-4797-3p, hsa-miR-665, lncRNA FBXL19-AS1/miR-378f/MRPL39 and lncRNA UBL7-AS1/miR-378f/MRPL39 as early clinical diagnosis and targeted therapy for DR. Achieve early clinical translation of research results.

Consistent with our view, many scholars have also investigated potential regulatory molecules in diabetic retinopathy (Shao et al., 2019; Wu et al., 2019; Zhu et al., 2021), and bioinformatics approaches have been employed in these studies. For example, Zhu et al. obtained DR-related lncRNAs and mRNAs from public databases and investigated the correlation between lncRNAs and mRNAs (Zhu et al., 2021). Wu et al. performed microarray sequencing of retinal samples from diabetic mice and constructed a ceRNA network based on the differentially expressed lncRNAs and mRNAs from the sequencing results (Wu et al., 2019); Shao et al. collected human retinal endothelial cells for transcript analysis and constructed 11 potential lncRNA/miRNA/mRNA networks (Shao et al., 2019).

However, it is worth mentioning that the present study still has many novelties compared with the published articles. First, this study utilized a dataset of retinal samples from DR patients for analysis, effectively avoiding data bias caused by species differences. Second, compared with the study by Wu and Shao et al. we further validated the predicted miRNAs and lncRNAs using the ncRNA dataset from human retinal samples, and constructed two ceRNA networks that are consistent with the ceRNA hypothesis and validated by the dataset. Finally, in contrast to the study by Zhu et al., this study correlated the target genes with their expression levels in DR patients, revealing the expression changes of the target gene MRPL39 in real DR pathological states and its integral lncRNA/miRNA/mRNA regulatory network. In Table 4, we exhibited the innovative features of this study compared with published articles.

**TABLE 4:** Some novelties in this study compared to published studies.

Items	Exploring the hub genes of diabetic retinopathy through bioinformatics analysis			
	Our findings	Cell paper (PMID: 34707685)	Cell paper (PMID: 30535492)	Cell paper (PMID: 31615521)
Years	2022	2021	2019	2019
Source of raw data	GSE140959 and GSE53257	Download from public resources	Gene expression microarray data in the retina of diabetic mice	Transcriptome profiling data of human retinal endothelial cells (HRECs)
Species/Tissue	Human/Plasma/Retina	−	Mice/Retina	Human/Retina
Hub Genes	IRHG: MRPL39	MIR4435-2HG	−	−
Validation set	GSE160308 and GSE160306	GSE102485	−	−
Verification	−	−	−	Human aqueous humor and serum: qRT-PCR (partial validation)
Mechanism	ceRNA network	lncRNA-TF	Co-expression network of lncRNA-miRNA-mRNA	11 TTR-lncRNA/miRNA/mRNA sub-networks
	lncRNA FBXL19-AS1/miR-378f/MRPL39	MIR4435-2HG/ERG		
	lncRNA UBL7-AS1/miR-378f/MRPL39	MIR4435-2HG/PPARG		

TF: transcript factors; TTR: transthyretin; qRT-PCR: quantitative reverse transcription polymerase chain reaction.

In conclusion, by bioinformatic analysis of microarray sequencing data from DR plasma and retinal samples, this study screened three potential miRNA biomarkers that can be applied as diagnostic for DR: hsa-miR-1179, hsa-miR-4797-3p and hsa-miR-665, and two ceRNA networks with immunomodulatory effects in DR: lncRNA FBXL19-AS1/miR-378f/MRPL39 and lncRNA UBL7-AS1/miR-378f/MRPL39. By exploring the combination of ncRNAs and mRNAs at the transcriptional level, this study shows a new aspect of DR mechanism regulation, which will provide promising theoretical support for the early clinical diagnosis and gene targeting therapy of DR. However, there are still some areas for further improvement in this study. This study is mainly based on bioinformatics methods to explore the mechanism of DR. Therefore, further combining clinical information and conducting experimental verification can further enhance the authenticity and credibility of this paper. In addition, the datasets used in the current study have a small sample size for analysis and validation, and the follow-up needs to continue to increase the sample size and conduct prospective cohort studies to further confirm our point of view.

## Data Availability

The datasets presented in this study can be found in online repositories. The names of the repository/repositories and accession number(s) can be found in the article/Supplementary Material.

## Supplementary material

Supplementary Table S1 GO/KEGG analysis results of cluster 1.

Supplementary Table S2 Results of miRNA prediction of MRPL39.

Supplementary Table S3 Results of the predicted lncRNA of hsa-miR-378f.

Supplementary Table S4 Results of the predicted lncRNA of hsa-miR-6849-5p.
